# The public health risks of counterfeit pills

**DOI:** 10.1016/S2468-2667(24)00273-1

**Published:** 2025-01

**Authors:** Joseph Friedman, Daniel Ciccarone

**Affiliations:** Department of Psychiatry, University of California, San Diego, San Diego, CA, USA (J Friedman MD); Department of Family Community Medicine, University of California, San Francisco, San Francisco, CA, USA (Prof D Ciccarone MD)

## Abstract

Synthetic illicit drugs, such as nitazenes and fentanyls, are becoming commonplace in countries around the world, including in Europe, Australia, and Latin America, which raises concern for overdose crises like those seen in North America. An important dimension of the risk represented by synthetic drugs is the fact that they are increasingly packaged in counterfeit pill form. These pills—often indistinguishable from authentic pharmaceuticals—have substantially widened the scope of populations susceptible to synthetic drug overdose in North America (eg, among adolescents experimenting with pills or tourists from the USA seeking psychoactive medications from pharmacies in Mexico). The non-medical use of diverted prescription medications is relatively more common, and less stigmatised, than the use of powder drugs. Many consumers of counterfeit pills are unaware that they contain synthetic illicit drugs, believe them to be authentic pharmaceuticals, and would be unlikely to consume those drugs knowingly or if in powder form. Given these issues, we recommend the expansion of educational and awareness campaigns, pill testing programmes to help consumers shift demand to safer products, increased monitoring in routine clinical scenarios and overdose death toxicology, and expanding medically managed safer alternatives to counterfeit pill use.

## Introduction

The proliferation of synthetic illicit drugs—especially fentanyl and fentanyl analogues—has driven a high rate of drug-related mortality in North America over the past decade. Over the past 5 years, a profound transition has occurred, with these substances increasingly prepackaged for sale in illicit markets as counterfeit pharmaceutical pills.^1^ The fraction of all illicit fentanyl seized by US law enforcement that was found in pill form rose from 10·3% in 2017 to 49·0% in 2023.^2^ The greater social acceptability and less stigma associated with pills compared with powder forms of drugs, such as heroin, has broadened the scope of populations potentially susceptible to using synthetic substances.^3,4^

In the USA and Canada, the so-called first wave of the overdose crisis began in the late 1990s with broad access to authentic prescription opioids through the health-care system and street-level diversion.^5^ This increased availability led to the development of a large cohort of individuals with opioid use disorder.^5^ The health-care and legal systems responded by broadly reducing access to legal opioids; federal and state policies mandated or strongly recommended rapid de-prescribing of opioids, and databases were implemented for tracking controlled substance prescriptions.^6^ These actions resulted in a substantial proportion of the opioid demand shifting to heroin obtained from illicit markets,^5^ and was followed by a rapid shift to heroin adulterated or substituted with illicit fentanyl,^7^ which paved the way for the substantial demand for fentanyl-based counterfeit pills.^8,9^ In each transition, individuals dependent on illicit opioids found themselves with little recourse but to switch their consumption patterns, as alternatives rapidly dwindled; for example, amid the takeover of fentanyl, heroin virtually disappeared from the illicit markets in many locations.

Counterfeit pill consumption has qualities distinct from previous waves of the crisis, as in many cases there is a mismatch between buyers’ demand for authentic pills and their receipt of (and subsequent exposure to) illicitly manufactured products. In our experience, even many consumers of illicit substances who are aware of the existence of counterfeit pills believe that their supply of pills is authentic, until presented with direct evidence to the contrary. Counterfeit medications circulating in North America are often impossible to distinguish with the naked eye from authentic versions of widely diverted medications, such as oxycodone, alprazolam, and stimulants. In other instances, counterfeit pills can be easily detected as inauthentic due to distinct texture, colouring, or other aspects of physical appearance. Although people who use drugs report that counterfeit pills are sometimes easy to spot, ultimately no pill or tablet obtained through an illicit market can be trusted as authentic without chemical analysis. Counterfeit pills have emerged in virtually all contexts where illicit drugs can be found, including on the dark web, from local and regional drug sellers, and traded between friends and acquaintances (who often believe the pills to be authentic prescriptions).^4,10–12^

As there are now signs that illicit synthetic drugs—often in counterfeit pill form—are emerging and driving overdoses in Europe, Australia, Latin America, and other locations globally,^13–16^ insights from North America’s experience with fake medications warrant consideration.

## Counterfeit pills expand the market for fentanyl and other synthetic drugs

A notable example of the demand-expanding effects of counterfeit pills can be found in increasing overdose deaths among adolescents in the USA. Between 2019 and 2020, the overdose death rate in adolescents aged 14–18 years in the USA doubled in a single year, and has continued to increase sharply; counterfeit pills are stongly linked by ecological evidence as the cause.^17^ Whereas only 0·3% of students aged 18 years report having ever used heroin, 5·0% report recreational use of prescription pills—a gap of more than 15-fold, which shows the role of stigma and cultural norms in shaping illicit substance consumption patterns.^3^ Another salient example is from ethnographic and drug checking data from Mexico, which have shown that tourists from the USA seeking medications such as oxycodone, alprazolam, and stimulants have been exposed to fentanyl, heroin, and methamphetamine-based counterfeit pills, sold directly from pharmacies that appear genuine but sell counterfeits.^12^ This activity appears to be restricted to independent, non-chain pharmacies, which have long catered to the clandestine practice of tourists seeking psychoactive medications without a prescription.^12^ Many of these tourists would probably never seek these same drugs sold in street powder form; however, the legitimacy of pharmaceutical products and establishments can be co-opted to assist the sale of illicit synthetic drugs when presented as pharmaceutical mimics.

In Canada, a robust market for diverted pharmaceuticals has also been widely infiltrated by synthetic mixtures. For example, of approximately 4000 pills tested as part of a drug checking programme in British Columbia between 2018 and 2024, nearly 50% contained unexpected substances.^18^ Notably, 20% of pills tested contained a benzodiazepine—including pharmaceutical benzodiazepines and novel synthetic benzodiazepines, such as etizolam, flualprazolam, and bromazolam—which considerably increase overdose risk when taken alongside synthetic opioids.^19^

## The possibility of a global synthetic drug crisis

Outside of North America, synthetic drug use has historically been low. However, in many countries, diverted pharmaceutical opioids, benzodiazepines, and other medications are commonly used recreationally and appear in overdose deaths.^20–22^ As diverted pills, obtained through illicit markets, are already relatively commonplace and less stigmatised, there could be a much broader potential consumer base for fentanyl-based counterfeit pills compared with heroin. There are also relatively few technical or logistical barriers to illicit synthetic opioid uptake in Europe and elsewhere. Europe already has sophisticated synthetic illicit drug production capacities; to date, these have primarily been focused on drugs such as MDMA,^23^ however, the technical expertise and access to precursor materials currently exist to produce synthetic opioids in Europe and many other world regions.^24^ There are also powerful economic incentives to switch from agricultural drug products such as cocaine and heroin, to synthetic products such as methamphetamine and cocaine, as they are generally substantially more profitable and easier to transport.^6,25^

Looking globally, the Taliban’s opium ban in Afghanistan is an important factor that could accelerate the uptake of synthetic drugs in Europe and other countries with entrenched heroin markets.^16,24^ To fill the void of dwindling heroin supplies, local synthesis of illicit fentanyls and other synthetic drugs, or their importation from Mexico, Estonia, or China (along with pill presses), are technologically and logistically realistic outcomes. Of note, Mexican drug trafficking organisations have gained inroads to the European illicit drug market.^26^

There are already signs that a shift towards illicit synthetic drugs is starting to occur worldwide.^13,15,27^ In the past several years, nitazenes (a family of potent synthetic opioids that are not approved for use in humans) have been detected in overdose outbreaks and illicit drug supplies (including counterfeit pills, in some cases) in the UK, Estonia, Latvia, France, and Ireland.^13,14^ Novel synthetic benzodiazepines are also increasingly prevalent in many countries across Europe. One such benzodiazepine—etizolam—is already present in two-thirds of overdose deaths in Scotland, and its prominence followed in the wake of crackdowns on access to prescription benzodiazepines diverted from the health-care system.^28^ The veterinary tranquilizer xylazine, which is also becoming commonplace in street drugs across the USA, has been found in counterfeit pills and vaporisers in the UK.^29^ Emerging markets for illicit fentanyls and nitazenes have also been found in countries in Latin America, including Brazil, Costa Rica, Peru, Chile, and Argentina.^27,30^ In Buenos Aires, cocaine laced with carfentanil led to a 24-fatality overdose outbreak in 2022.^31^ In Australia, both fentanyl and nitazenes have emerged in numerous overdose outbreaks over the past decade, although, to date, none of these overdose outbreaks have been as sustained or as large in magnitude as trends seen in North America.^15,32–34^ Across these emerging markets for synthetic illicit drugs, a large number of consumers of diverted pharmaceutical medications could be caught off guard by the rise of counterfeit pills.

## Recommendations to reduce harms from counterfeit pills

As illicit synthetic drug distribution, use, and overdose are on the rise in numerous countries worldwide, their proliferation in counterfeit pills is a crucial dimension for consideration. We recommend the following strategies, which could counter the harms of unintentional exposure to illicit synthetic drugs.

### Broad education about counterfeit pill risk

Primary prevention can have a more prominent role with counterfeit pills compared with other drugs, as considerable risk comes from consumers being unaware of their inauthentic status. Public health agencies could avert harm by quickly informing potential consumers about the dangers of emerging counterfeit products containing synthetic drugs. Given the particular risk of overdoses involving counterfeit pills among adolescents and young people in North America, schools, colleges, and universities could serve as fruitful locations for interventions.^35,36^

### Pill testing services

Drug checking services are a useful form of secondary prevention, as they use point-of-care technologies to describe the contents of drug samples, empowering consumers to shift demand away from more dangerous or counterfeit products.^37^ These programmes range from mail-in services (for which consumers send in samples anonymously and results are posted online), to community-facing services that provide results to consumers in real time. For maximum effect, we suggest that client-facing services are implemented widely in spaces frequented by people who use drugs and could be at risk of overdose—such as music venues, club scenes, harm reduction centres, and syringe exchanges—to provide detailed drug checking results alongside tailored counselling. These efforts are supported by advances in portable drug checking technologies, which are becoming less expensive and more capable of providing detailed qualitative and quantitative results.

Drug checking technologies can also inform physicians and public health professionals which emerging drugs should be routinely tested for. However, these services are often less accessible for consumers who are living in poverty or who face higher levels of criminalisation from law enforcement. Therefore testing services must be implemented in a manner that intentionally reaches groups more susceptible to overdose risk.^38,39^ Additionally, testing services might not decrease overdose risk for some consumers if they have opioid dependence and no safer options are present. Nevertheless, for those consumers of drugs who are able to shift or modify their demand, drug checking allows individuals to prioritise safer options.

### Increase public health monitoring of counterfeit pill contents

Given the rapidly evolving nature of the illicit drug supply—with new drugs and combinations constantly being developed and sold—it is an open question if public health monitoring will be able to keep up. Renewed investments are needed in supply-side monitoring efforts to detect new substances, sources, and polysubstance formulations that are being used, and trends in usage. These monitoring efforts must be linked to communication networks that broadcast news about emerging risks to consumers. Encouraging efforts to put early warning alert networks into place and bolster public health monitoring of synthetic drug crises are ongoing, for example, through the European Union Drugs Agency’s Early Warning System for Novel Psychoactive Substances^40^ and the Organization of American States’ Early Alert System for the Americas.^27,30^ To best monitor for emerging threats, these systems should ideally leverage and integrate a broad range of supply-side indicators, including data from law enforcement drug seizures^1,41^ (which must be made rapidly available to the public and researchers) alongside community-engaged drug checking services,^37^ routinely collected clinical indicators from pre-hospital and emergency department encounters involving fatal and non-fatal overdose events,^42^ and rapid toxicology from overdose autopsy investigations leveraging panels of tests for novel synthetic substances.

### Avoid crackdowns on diverted (authentic) pharmaceutical products until safe replacement options are widely available

Data from North America, and other nations around the world, highlight that reductions in access to prescription medications, including the illicit use of diverted medications, must be done with careful attention to where demand for these products might be displaced.^24^ Demand shifting onto counterfeit pills represents a high-risk outcome. In the best-case scenario, demand is shifted to safe, medically managed products and health-care services, such as buprenorphine and methadone. There is also long-standing and growing evidence supporting the use of a broader array of medically managed substitution therapy options (including hydromorphone, diacetylmorphine, and morphine).^43,44^ Nevertheless, legal and regulatory restrictions mean these options remain limited for most patients globally, and are almost entirely restricted in many countries, such as the USA. There are ongoing trials and pilot programmes of community-based safer supply programmes in Canada, providing access to opioid maintenance through a range of partnerships between medical personnel and harm reduction and community organisations, some of which have shown promising reductions in overdose mortality.^45–51^

Governments have used various approaches to try to reduce the supply of synthetic drugs and their precursors, with mixed success and consequences.^52,53^ We urge decision makers to be thoughtful about the costs of these strategies, as entrenched demand will often shift to higher risk options amidst crackdowns, unless safer options are made readily available.^24^ Additionally, it is essential to note that fentanyl and fentanyl analogues are necessary medications used safely for various medical purposes, and efforts to curb access should be limited to illicit opioids.

## Conclusions

Given the troubling global trajectory of synthetic drug use, and the concerning uncertainty of how severe the situation will become, there is value in public health practitioners, clinicians, and policy makers considering the properties of counterfeit pills that expand the scope of populations susceptible to synthetic drug overdose. Counterfeit pills can expose a broader segment of consumers to unintentional illicit synthetic drug use, which necessitates a swift and tailored public health response.
